# Supplementary material for: Dynalign II: common secondary structure prediction for RNA homologs with domain insertions
Source: Nucleic Acids Res. 2014 Nov 21;42(22):13939–48. doi: 10.1093/nar/gku1172 (PMC4267632; doi:10.1093/nar/gku1172)
Supplement: SUPPLEMENTARY DATA [file supp_gku1172_nar-02021-z-2014-File012.zip › manual/javadoc/RNAstructure_java_interface/source/windows/OligoWalkWindow.StringComboBox.html]

OligoWalkWindow.StringComboBox


JavaScript is disabled on your browser.


- Overview
- Package
- Class
- Tree
- Index
- Help

- Prev Class
- Next Class

- Frames
- No Frames

- All Classes

- Summary:
- Nested |
- Field |
- Constr |
- Method

- Detail:
- Field |
- Constr |
- Method


RNAstructure\_java\_interface.source.windows

## Class OligoWalkWindow.StringComboBox

- java.lang.Object
- - java.awt.Component
  - - java.awt.Container
    - - javax.swing.JComponent
      - - javax.swing.JComboBox
        - - RNAstructure\_java\_interface.source.windows.OligoWalkWindow.StringComboBox

- All Implemented Interfaces:
  :   ActionListener, ImageObserver, ItemSelectable, MenuContainer, Serializable, EventListener, Accessible, ListDataListener

  Enclosing class:
  :   OligoWalkWindow

  ---

    

  ```
  public class OligoWalkWindow.StringComboBox
  extends JComboBox
  ```

  An inner class that creates a combo box holding a string list.
    
    
  Note that this class is compatible with Java 6, but not fully compatible
  with Java 7. Java 6 does not require JComboBox to be parameterized, but
  Java 7 does. Raw type warnings and unchecked type warnings are
  suppressed in this class to ensure backward compatibility.

  See Also:
  :   Serialized Form

- - ### Nested Class Summary

    - ### Nested classes/interfaces inherited from class javax.swing.JComboBox

      `JComboBox.AccessibleJComboBox, JComboBox.KeySelectionManager`
    - ### Nested classes/interfaces inherited from class javax.swing.JComponent

      `JComponent.AccessibleJComponent`
    - ### Nested classes/interfaces inherited from class java.awt.Container

      `Container.AccessibleAWTContainer`
    - ### Nested classes/interfaces inherited from class java.awt.Component

      `Component.AccessibleAWTComponent, Component.BaselineResizeBehavior, Component.BltBufferStrategy, Component.FlipBufferStrategy`
  - ### Field Summary

    Fields

    | Modifier and Type | Field and Description |
    | --- | --- |
    | `private static long` | `serialVersionUID` |

    - ### Fields inherited from class javax.swing.JComboBox

      `actionCommand, dataModel, editor, isEditable, keySelectionManager, lightWeightPopupEnabled, maximumRowCount, renderer, selectedItemReminder`
    - ### Fields inherited from class javax.swing.JComponent

      `accessibleContext, listenerList, TOOL_TIP_TEXT_KEY, ui, UNDEFINED_CONDITION, WHEN_ANCESTOR_OF_FOCUSED_COMPONENT, WHEN_FOCUSED, WHEN_IN_FOCUSED_WINDOW`
    - ### Fields inherited from class java.awt.Component

      `BOTTOM_ALIGNMENT, CENTER_ALIGNMENT, LEFT_ALIGNMENT, RIGHT_ALIGNMENT, TOP_ALIGNMENT`
    - ### Fields inherited from interface java.awt.image.ImageObserver

      `ABORT, ALLBITS, ERROR, FRAMEBITS, HEIGHT, PROPERTIES, SOMEBITS, WIDTH`
  - ### Constructor Summary

    Constructors

    Constructor and Description

    `OligoWalkWindow.StringComboBox(String... choices)`

    Constructor.
  - ### Method Summary

    Methods

    | Modifier and Type | Method and Description |
    | --- | --- |
    | `String` | `getSelectedItem()` |

    - ### Methods inherited from class javax.swing.JComboBox

      `actionPerformed, actionPropertyChanged, addActionListener, addItem, addItemListener, addPopupMenuListener, configureEditor, configurePropertiesFromAction, contentsChanged, createActionPropertyChangeListener, createDefaultKeySelectionManager, fireActionEvent, fireItemStateChanged, firePopupMenuCanceled, firePopupMenuWillBecomeInvisible, firePopupMenuWillBecomeVisible, getAccessibleContext, getAction, getActionCommand, getActionListeners, getEditor, getItemAt, getItemCount, getItemListeners, getKeySelectionManager, getMaximumRowCount, getModel, getPopupMenuListeners, getPrototypeDisplayValue, getRenderer, getSelectedIndex, getSelectedObjects, getUI, getUIClassID, hidePopup, insertItemAt, installAncestorListener, intervalAdded, intervalRemoved, isEditable, isLightWeightPopupEnabled, isPopupVisible, paramString, processKeyEvent, removeActionListener, removeAllItems, removeItem, removeItemAt, removeItemListener, removePopupMenuListener, selectedItemChanged, selectWithKeyChar, setAction, setActionCommand, setEditable, setEditor, setEnabled, setKeySelectionManager, setLightWeightPopupEnabled, setMaximumRowCount, setModel, setPopupVisible, setPrototypeDisplayValue, setRenderer, setSelectedIndex, setSelectedItem, setUI, showPopup, updateUI`
    - ### Methods inherited from class javax.swing.JComponent

      `addAncestorListener, addNotify, addVetoableChangeListener, computeVisibleRect, contains, createToolTip, disable, enable, firePropertyChange, firePropertyChange, firePropertyChange, fireVetoableChange, getActionForKeyStroke, getActionMap, getAlignmentX, getAlignmentY, getAncestorListeners, getAutoscrolls, getBaseline, getBaselineResizeBehavior, getBorder, getBounds, getClientProperty, getComponentGraphics, getComponentPopupMenu, getConditionForKeyStroke, getDebugGraphicsOptions, getDefaultLocale, getFontMetrics, getGraphics, getHeight, getInheritsPopupMenu, getInputMap, getInputMap, getInputVerifier, getInsets, getInsets, getListeners, getLocation, getMaximumSize, getMinimumSize, getNextFocusableComponent, getPopupLocation, getPreferredSize, getRegisteredKeyStrokes, getRootPane, getSize, getToolTipLocation, getToolTipText, getToolTipText, getTopLevelAncestor, getTransferHandler, getVerifyInputWhenFocusTarget, getVetoableChangeListeners, getVisibleRect, getWidth, getX, getY, grabFocus, isDoubleBuffered, isLightweightComponent, isManagingFocus, isOpaque, isOptimizedDrawingEnabled, isPaintingForPrint, isPaintingOrigin, isPaintingTile, isRequestFocusEnabled, isValidateRoot, paint, paintBorder, paintChildren, paintComponent, paintImmediately, paintImmediately, print, printAll, printBorder, printChildren, printComponent, processComponentKeyEvent, processKeyBinding, processMouseEvent, processMouseMotionEvent, putClientProperty, registerKeyboardAction, registerKeyboardAction, removeAncestorListener, removeNotify, removeVetoableChangeListener, repaint, repaint, requestDefaultFocus, requestFocus, requestFocus, requestFocusInWindow, requestFocusInWindow, resetKeyboardActions, reshape, revalidate, scrollRectToVisible, setActionMap, setAlignmentX, setAlignmentY, setAutoscrolls, setBackground, setBorder, setComponentPopupMenu, setDebugGraphicsOptions, setDefaultLocale, setDoubleBuffered, setFocusTraversalKeys, setFont, setForeground, setInheritsPopupMenu, setInputMap, setInputVerifier, setMaximumSize, setMinimumSize, setNextFocusableComponent, setOpaque, setPreferredSize, setRequestFocusEnabled, setToolTipText, setTransferHandler, setUI, setVerifyInputWhenFocusTarget, setVisible, unregisterKeyboardAction, update`
    - ### Methods inherited from class java.awt.Container

      `add, add, add, add, add, addContainerListener, addImpl, addPropertyChangeListener, addPropertyChangeListener, applyComponentOrientation, areFocusTraversalKeysSet, countComponents, deliverEvent, doLayout, findComponentAt, findComponentAt, getComponent, getComponentAt, getComponentAt, getComponentCount, getComponents, getComponentZOrder, getContainerListeners, getFocusTraversalKeys, getFocusTraversalPolicy, getLayout, getMousePosition, insets, invalidate, isAncestorOf, isFocusCycleRoot, isFocusCycleRoot, isFocusTraversalPolicyProvider, isFocusTraversalPolicySet, layout, list, list, locate, minimumSize, paintComponents, preferredSize, printComponents, processContainerEvent, processEvent, remove, remove, removeAll, removeContainerListener, setComponentZOrder, setFocusCycleRoot, setFocusTraversalPolicy, setFocusTraversalPolicyProvider, setLayout, transferFocusDownCycle, validate, validateTree`
    - ### Methods inherited from class java.awt.Component

      `action, add, addComponentListener, addFocusListener, addHierarchyBoundsListener, addHierarchyListener, addInputMethodListener, addKeyListener, addMouseListener, addMouseMotionListener, addMouseWheelListener, bounds, checkImage, checkImage, coalesceEvents, contains, createImage, createImage, createVolatileImage, createVolatileImage, disableEvents, dispatchEvent, enable, enableEvents, enableInputMethods, firePropertyChange, firePropertyChange, firePropertyChange, firePropertyChange, firePropertyChange, firePropertyChange, getBackground, getBounds, getColorModel, getComponentListeners, getComponentOrientation, getCursor, getDropTarget, getFocusCycleRootAncestor, getFocusListeners, getFocusTraversalKeysEnabled, getFont, getForeground, getGraphicsConfiguration, getHierarchyBoundsListeners, getHierarchyListeners, getIgnoreRepaint, getInputContext, getInputMethodListeners, getInputMethodRequests, getKeyListeners, getLocale, getLocation, getLocationOnScreen, getMouseListeners, getMouseMotionListeners, getMousePosition, getMouseWheelListeners, getName, getParent, getPeer, getPropertyChangeListeners, getPropertyChangeListeners, getSize, getToolkit, getTreeLock, gotFocus, handleEvent, hasFocus, hide, imageUpdate, inside, isBackgroundSet, isCursorSet, isDisplayable, isEnabled, isFocusable, isFocusOwner, isFocusTraversable, isFontSet, isForegroundSet, isLightweight, isMaximumSizeSet, isMinimumSizeSet, isPreferredSizeSet, isShowing, isValid, isVisible, keyDown, keyUp, list, list, list, location, lostFocus, mouseDown, mouseDrag, mouseEnter, mouseExit, mouseMove, mouseUp, move, nextFocus, paintAll, postEvent, prepareImage, prepareImage, processComponentEvent, processFocusEvent, processHierarchyBoundsEvent, processHierarchyEvent, processInputMethodEvent, processMouseWheelEvent, remove, removeComponentListener, removeFocusListener, removeHierarchyBoundsListener, removeHierarchyListener, removeInputMethodListener, removeKeyListener, removeMouseListener, removeMouseMotionListener, removeMouseWheelListener, removePropertyChangeListener, removePropertyChangeListener, repaint, repaint, repaint, resize, resize, setBounds, setBounds, setComponentOrientation, setCursor, setDropTarget, setFocusable, setFocusTraversalKeysEnabled, setIgnoreRepaint, setLocale, setLocation, setLocation, setName, setSize, setSize, show, show, size, toString, transferFocus, transferFocusBackward, transferFocusUpCycle`
    - ### Methods inherited from class java.lang.Object

      `clone, equals, finalize, getClass, hashCode, notify, notifyAll, wait, wait, wait`

- - ### Field Detail


    - #### serialVersionUID

      ```
      private static final long serialVersionUID
      ```

      See Also:
      :   Constant Field Values
  - ### Constructor Detail


    - #### OligoWalkWindow.StringComboBox

      ```
      public OligoWalkWindow.StringComboBox(String... choices)
      ```

      Constructor.

      Parameters:
      :   `choices` - The choices available in this box.
  - ### Method Detail


    - #### getSelectedItem

      ```
      public String getSelectedItem()
      ```

      **Overrides:**
      :   `getSelectedItem` in class `JComboBox`


- Overview
- Package
- Class
- Tree
- Index
- Help

- Prev Class
- Next Class

- Frames
- No Frames

- All Classes

- Summary:
- Nested |
- Field |
- Constr |
- Method

- Detail:
- Field |
- Constr |
- Method
